# Different prion disease phenotypes result from inoculation of cattle with two temporally separated sources of sheep scrapie from Great Britain

**DOI:** 10.1186/1746-6148-2-31

**Published:** 2006-10-17

**Authors:** Timm Konold, Yoon Hee Lee, Michael J Stack, Claire Horrocks, Robert B Green, Melanie Chaplin, Marion M Simmons, Steve AC Hawkins, Richard Lockey, John Spiropoulos, John W Wilesmith, Gerald AH Wells

**Affiliations:** 1 Veterinary Laboratories Agency Weybridge, New Haw, Addlestone, UK; 2 National Veterinary Research and Quarantine Service, Anyang, Republic of Korea; 3 Department for Environment, Food and Rural Affairs, 1A Page Street, London, UK

## Abstract

**Background:**

Given the theoretical proposal that bovine spongiform encephalopathy (BSE) could have originated from sheep scrapie, this study investigated the pathogenicity for cattle, by intracerebral (i.c.) inoculation, of two pools of scrapie agents sourced in Great Britain before and during the BSE epidemic.

Two groups of ten cattle were each inoculated with pools of brain material from sheep scrapie cases collected prior to 1975 and after 1990. Control groups comprised five cattle inoculated with sheep brain free from scrapie, five cattle inoculated with saline, and for comparison with BSE, naturally infected cattle and cattle i.c. inoculated with BSE brainstem homogenate from a parallel study. Phenotypic characterisation of the disease forms transmitted to cattle was conducted by morphological, immunohistochemical, biochemical and biological methods.

**Results:**

Disease occurred in 16 cattle, nine inoculated with the pre-1975 inoculum and seven inoculated with the post-1990 inoculum, with four cattle still alive at 83 months post challenge (as at June 2006). The different inocula produced predominantly two different disease phenotypes as determined by histopathological, immunohistochemical and Western immunoblotting methods and biological characterisation on transmission to mice, neither of which was identical to BSE. Whilst the disease presentation was uniform in all scrapie-affected cattle of the pre-1975 group, the post-1990 inoculum produced a more variable disease, with two animals sharing immunohistochemical and molecular profile characteristics with animals in the pre-1975 group.

**Conclusion:**

The study has demonstrated that cattle inoculated with different pooled scrapie sources can develop different prion disease phenotypes, which were not consistent with the phenotype of BSE of cattle and whose isolates did not have the strain typing characteristics of the BSE agent on transmission to mice.

## Background

Bovine spongiform encephalopathy (BSE) is a progressive and fatal neurological disease of cattle, which was first discovered in Great Britain (GB) in 1986 [1]. Epidemiological studies support the view that the cattle population was exposed to a scrapie-like agent through the feeding of meat and bone meal after changes in rendering practices. Recycling of the agent increased exposure of cattle to a scrapie-like agent, resulting in the large epidemic of BSE. It is hypothesised that the cattle-adapted BSE agent originated either from sheep scrapie, the transmission resulting directly in clinical disease, or from cattle with a scrapie-like disease that was present endemically in cattle at a low level prior to the epidemic [2].

Strain typing of transmissible spongiform encephalopathy (TSE) isolates in mice from cattle, exotic ruminant species and domestic cats, also of isolates of experimental BSE in sheep, goats and pigs [3,4] indicate the unique singularity of the BSE agent in GB, its apparent dissimilarity to isolates of natural scrapie of sheep, and its stability on natural or experimental single passage in the several species. Neuropathological studies profiling vacuolar changes in BSE [5,6] have also provided evidence of the single phenotype of BSE in GB and allow comparisons to be made with possible other TSE phenotypes in cattle caused by different agent strains.

As well as vacuolation within the brain, all TSEs are characterised by the accumulation of abnormal prion protein (PrP^Sc^) in the central nervous system. Synthetic forms of this protein may transmit disease phenotypes without utilising a nucleic acid genome by acting as a template, binding, and changing the conformation of the normal cellular prion protein (PrP^C^) to replicates of PrP^Sc ^[7]. Although both have the same amino-acid sequence, PrP^Sc^, unlike PrP^C^, is relatively resistant to proteolysis and insoluble in mild detergents. These different physico-chemical properties between normal and disease-associated PrP can be used to study the pathology and biochemistry of the disease group by the detection of PrP^Sc ^by immunohistochemistry (IHC), and the protease-resistant core of PrP^Sc ^(PrP^res^) by Western immunoblotting (WB). WB methods have recently been used to characterise different TSE strains, based on molecular masses and ratio of the resultant glycoforms after digestion with proteinase K [8]. These have shown similarities in different BSE isolates [8-10], which further suggests that BSE is caused by a single strain.

Experimental studies conducted in the USA have demonstrated that natural ovine scrapie can be transmitted to cattle after parenteral inoculation resulting in a prion disease with clinical and pathological findings unlike those of BSE [11,12] and that, on passage in cattle, the disease phenotype does not change substantially [13,14]. The results cannot be extrapolated to the situation in GB because of the potential difference between scrapie strains in the USA and in GB. To provide some understanding of the strain (or strains) of scrapie in sheep in GB, their possible changed properties on passage in cattle and the dynamics of a selection process which might result in BSE, a large series of studies were initiated in the United Kingdom in the mid 1990s. One of these, the present study, investigated the pathogenicity for cattle, by intracerebral (i.c.) inoculation, of two pools of scrapie agents sourced in GB before and during the BSE epidemic. The transmitted disease forms in recipient cattle were characterised by clinical, pathological, biochemical and biological strain typing approaches and compared with i.c. induced and naturally infected cases of BSE in cattle. This study design could, within the limitations of the source materials, potentially identify an endemic form of scrapie pathogenic for cattle and/or the occurrence of the BSE agent in the GB sheep population.

## Results

At the time of writing (June 2006), nine out of ten cattle from the pre-1975 group and seven out of ten cattle from the post-1990 group have succumbed to experimental challenge. The remaining live animals are currently at 83 months post inoculation (mpi, this is based on the calendar month and always rounded down).

### Clinical assessments

Two clinical presentations could be distinguished: a dull or somnolent form, exhibited by all nine steers in the pre-1975 group and by five steers in the post-1990 group, and a nervous form, exhibited by two animals (PG 920/01 and PG512/02) in the post-1990 group (Table 1). All affected steers became ataxic resulting in difficulty in rising. Steers presenting with the dull form stood motionless in the pen with the head lowered or resting against objects, such as hurdles or a wall, when undisturbed and did not over-react to external stimuli. However, this was not the case in the two steers presenting with the nervous form; these animals displayed over-reactivity to external stimuli and increased nervousness as the disease progressed. In addition, four animals in the pre-1975 group displayed signs suggestive of generalised seizures. These were found in lateral recumbency with limb "paddling", but regained their footing with assistance, although they appeared to have a slightly disoriented expression.

The times from inoculation to display of evident neurological signs suggestive of a prion disease (definite signs of a TSE, usually ataxia and dullness or nervousness) ranged from 18 to 54 months in both groups (mean and standard error of mean [SEM] 27.2 m and 3.6 m in the pre-1975 group and 30.1 m and 4.6 m in the post-1990 group, respectively). The mean incubation periods in both groups did not differ significantly (Mann-Whitney *U *test: p = 0.6).

One control steer inoculated with saline solution developed boisterous behaviour that compromised staff safety and was culled at 60 mpi. The behaviour was explained by the finding of some remaining testicular tissue on gross necropsy. Remaining control animals (four steers inoculated with saline solution and all five steers inoculated with TSE-free brain) are still alive and were clinically unremarkable as at June 2006.

### Histopathology and immunohistochemistry

Histopathological changes of a spongiform encephalopathy were present in the brains of all animals in which clinical neurological signs were observed.

**Figure 1:**
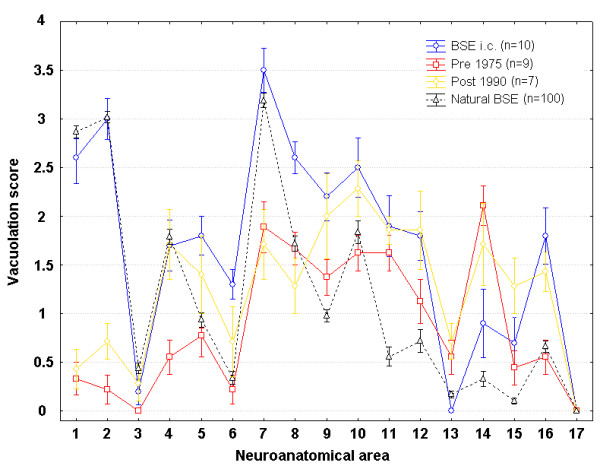
**Lesion profiles in cattle challenged with scrapie and comparison with BSE cases**. 1. Nucleus of the solitary tract. 2. Nucleus of the spinal tract of the trigeminal nerve. 3. Hypoglossal nucleus. 4. Vestibular nuclear complex. 5. Cochlear nucleus. 6. Cerebellar vermis. 7. Central grey matter. 8. Rostral colliculus. 9. Medial geniculate nucleus. 10. Hypothalamus. 11. Nucleus dorsomedialis thalami. 12. Nucleus ventralis lateralis thalami. 13. Frontal cortex. 14. Septal nuclei. 15. Caudate nucleus. 16. Putamen. 17. Claustrum.

**Figure 2:**
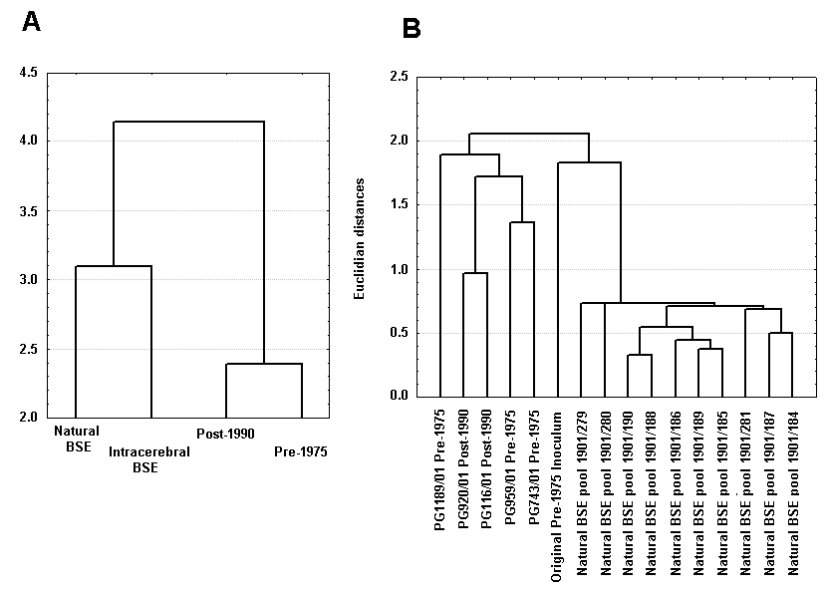
**Tree diagram after cluster analysis of the vacuolation scores in selected neuroanatomical brain areas**. A) Comparison of the profiles in cattle. B) Comparison of the profiles in mice. Natural BSE pool inoculum codes: study/inoculum.

The lesion profiles of both groups inoculated with scrapie material and comparisons with corresponding data from brains of cattle i.c. and naturally infected with BSE are given in Figure 1. Both scrapie inocula appear to produce a similar lesion profile in cattle with similar vacuolation scores in most of the 17 neuroanatomical areas and only minor differences (reduced vacuolation scores in vestibular nucleus complex, cochlear nucleus, caudate nucleus and putamen in the pre-1975 group). The most significant differences from natural and experimental (i.c.) BSE were observed in areas 1, 2 and 7 (markedly decreased vacuolation scores in the nucleus of the solitary tract, the spinal tract nucleus of the trigeminal nerve and central grey matter). Cluster analysis separated the pre-1975 and post-1990 group from natural and experimental BSE (Figure 2A).

**Figure 3:**
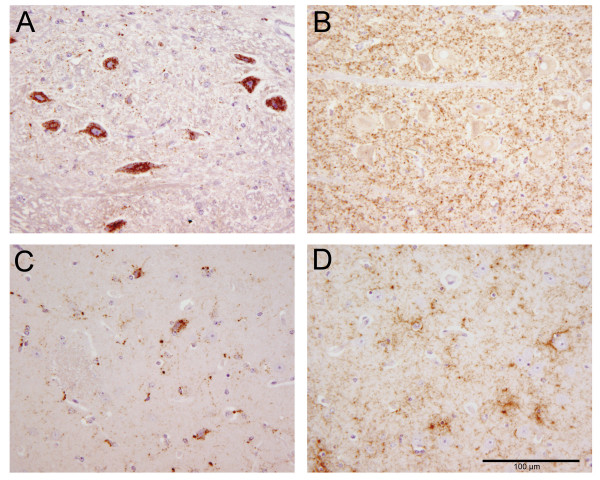
**Different PrP^Sc ^immunolabelling in the pre-1975 and post-1990 group**. A) Olivary nucleus, pre-1975 pool. Immunolabelling is predominantly intraneuronal – Group 1 type. B) Olivary nucleus, post-1990 pool. Immunolabelling is predominantly in the neuropil – Group 2 type. C) Caudate nucleus, pre-1975 pool. Immunolabelling is predominantly intracellular (intraneuronal and intraglial) – Group 1 type. D) Caudate nucleus, post-1990 pool. Immunolabelling is predominantly in the neuropil, with prominent stellate forms – Group 3 type.

By contrast the immunohistochemical examinations revealed three distinct patterns based on the type of PrP deposits seen in different neuroanatomical areas (Table 1). In brains from cattle challenged with the pre-1975 inoculum disease-specific accumulation was generally present throughout the central nervous system (CNS) and consisted of predominantly granular cytoplasmic PrP accumulation in neuronal perikarya (Figure 3A, C) and intra-glial deposits, although in one animal PrP accumulation was absent in the rostral medulla and the cerebellum. By contrast the post-1990 inoculum produced a widespread disease-specific PrP accumulation that consisted predominantly of particulate PrP accumulation in the neuropil (Figure 3B, D), accompanied by granular cytoplasmic PrP deposits. However, in two cases from the post-1990 group (PG 152/02 and 512/02), the pattern more closely resembled that of the pre-1975 group with the exception of cortical areas in which patterns similar to that of the post-1990 group were seen.

### Western blotting of PrP^res^

All diagnostic examinations of the saline inoculated steer culled at 60 mpi were negative.

**Figure 4:**
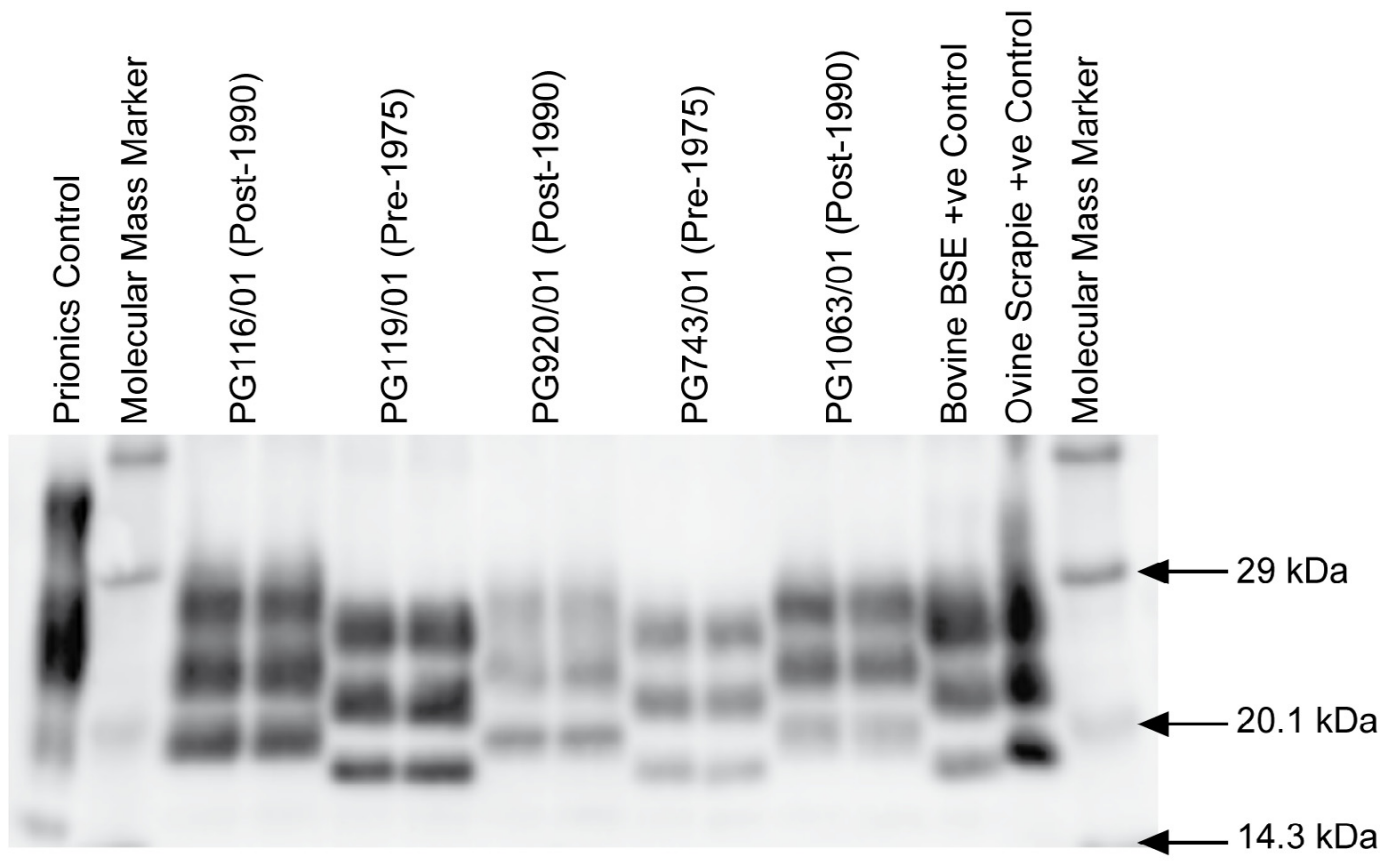
Western immunoblot with monoclonal antibody 6H4 on caudal medulla samples from cattle in the pre-1975 and post-1990 group.

**Figure 5:**
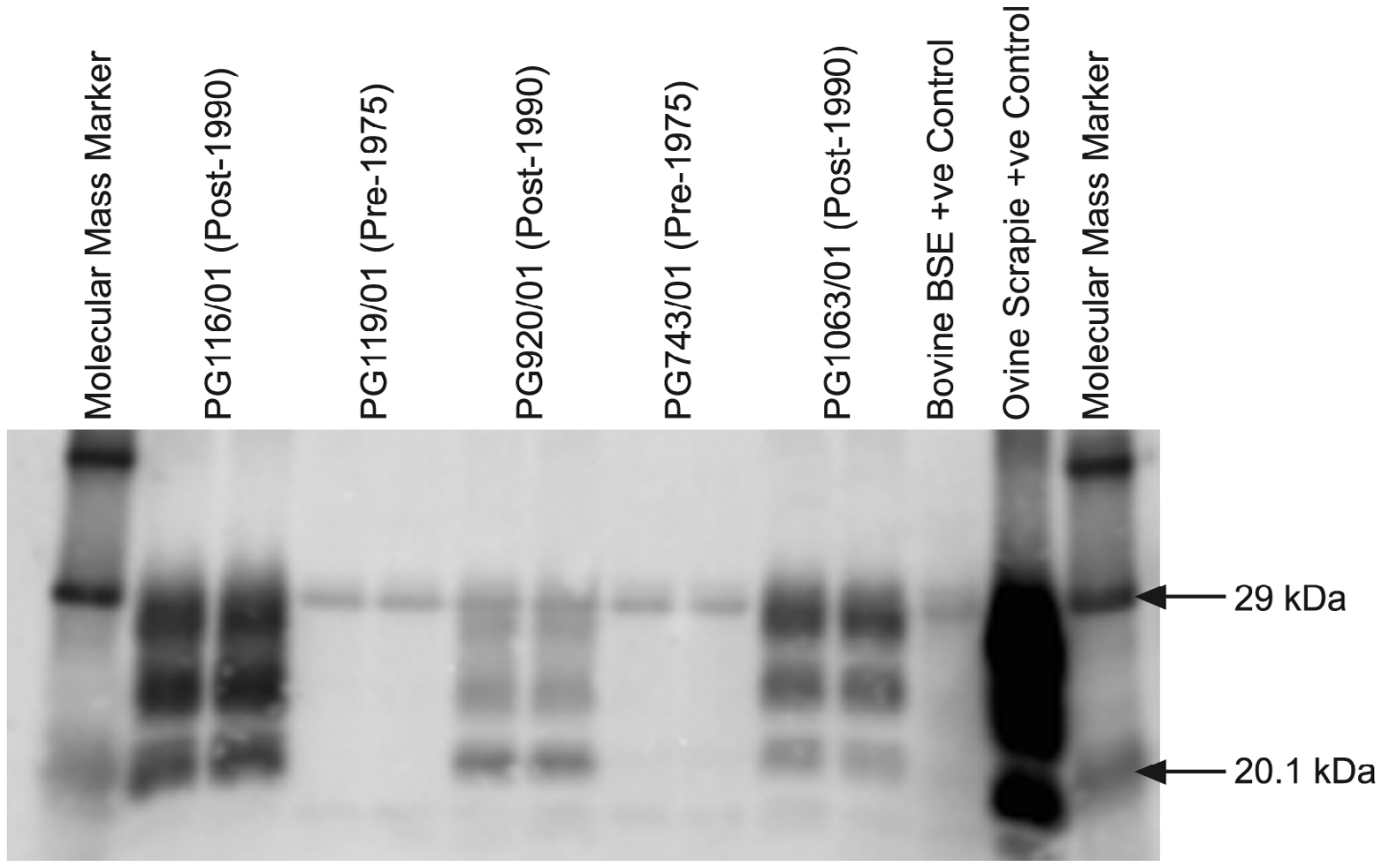
Western immunoblot with monoclonal antibody P4 on caudal medulla samples from cattle in the pre-1975 and post-1990 group.

All clinically affected cattle inoculated with scrapie tested positive for PrP^res^. However, the molecular profiles obtained with the VLA hybrid WB technique differed between the two groups, and none of the molecular profiles obtained were identical to the scrapie or BSE controls (Figures 4 and 5). Immunoblotting of caudal medulla from the eight pre-1975 challenged cattle gave a uniform molecular profile for all animals in the group. Using monoclonal antibody (mAb) 6H4, an unglycosylated band of lower molecular mass than the natural scrapie control sample was obtained and with mAb P4 there was no immuno-reaction. This was similar to the corresponding molecular profile obtained with these two mAbs for the natural cattle BSE control sample. However, although the molecular mass of the unglycosylated band was not significantly lower than that of the BSE control (approximately 0.2 kDa; p = 0.211) the monoglycosylated band (approximately 0.3 kDa; p = 0.008) and the diglycosylated band (approximately 0.5 kDa; p = 0.001) were significantly lower.

In contrast, the molecular profiles obtained from the seven cattle in the post-1990 group were more varied. The caudal medulla tissue of five clinically affected cattle gave a profile which had an unglycosylated band of higher molecular mass than the natural scrapie sample (approximately 1.5 kDa; p = 0.001), and this was approximately 1.7 kDa higher than that obtained for the BSE control sample. A marked immuno-reaction was obtained when mAb P4 was used on the caudal medulla from these five cattle. Frontal cortex and brainstem regions were also tested for one of these five cattle and the molecular profile remained the same (results not shown).

**Figure 6:**
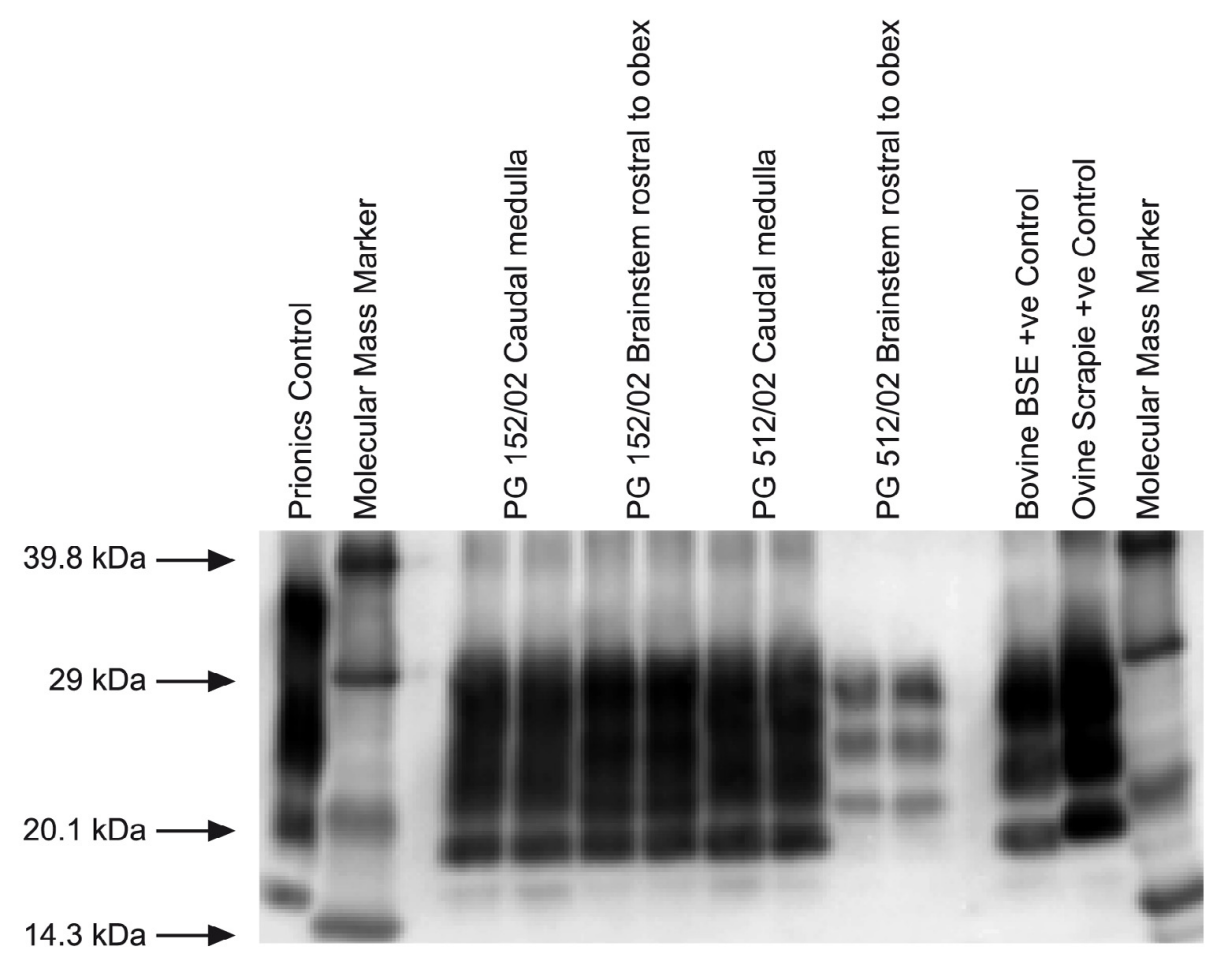
Western immunoblot with monoclonal antibody 6H4 on two different brain samples in outliers PG152/02 and PG512/02.

The caudal medulla from the remaining two steers from the post-1990 group (PG152/02 and PG512/02) gave a similar result to the other five animals but the unglycosylated protein band had a lower molecular mass. This differed only slightly from the cattle BSE control sample with the unglycosylated protein band approximately 0.5 kDa lower than that of the control. A sample of the brainstem, taken slightly more rostral to the obex, gave a WB profile in steer PG512/02 that resembled the profile of the other five steers (i.e. showing a higher molecular mass for the unglycosylated band with mAb 6H4), whilst it remained the same (i.e. lower molecular mass) in the other steer (PG152/02) (Figure 6). However, the mAb P4 immuno-reaction in all seven cattle from the post 1990 group remained evident regardless of brain region. Mean values for the unglycosylated, monoglycosylated and diglycosylated molecular masses are shown in Table 2. The means for the post-1990 samples were significantly higher than those for both control samples (p < 0.001). The pre-1975 sample means, with a single exception, were significantly lower than both control means. The means for the pre-1975 group of samples were significantly lower than those for the post-1990 group (p < 0.001). The average differences and their 95% confidence intervals are shown in Table 3 (excluding the outliers in the post-1990 group).

**Figure 7:**
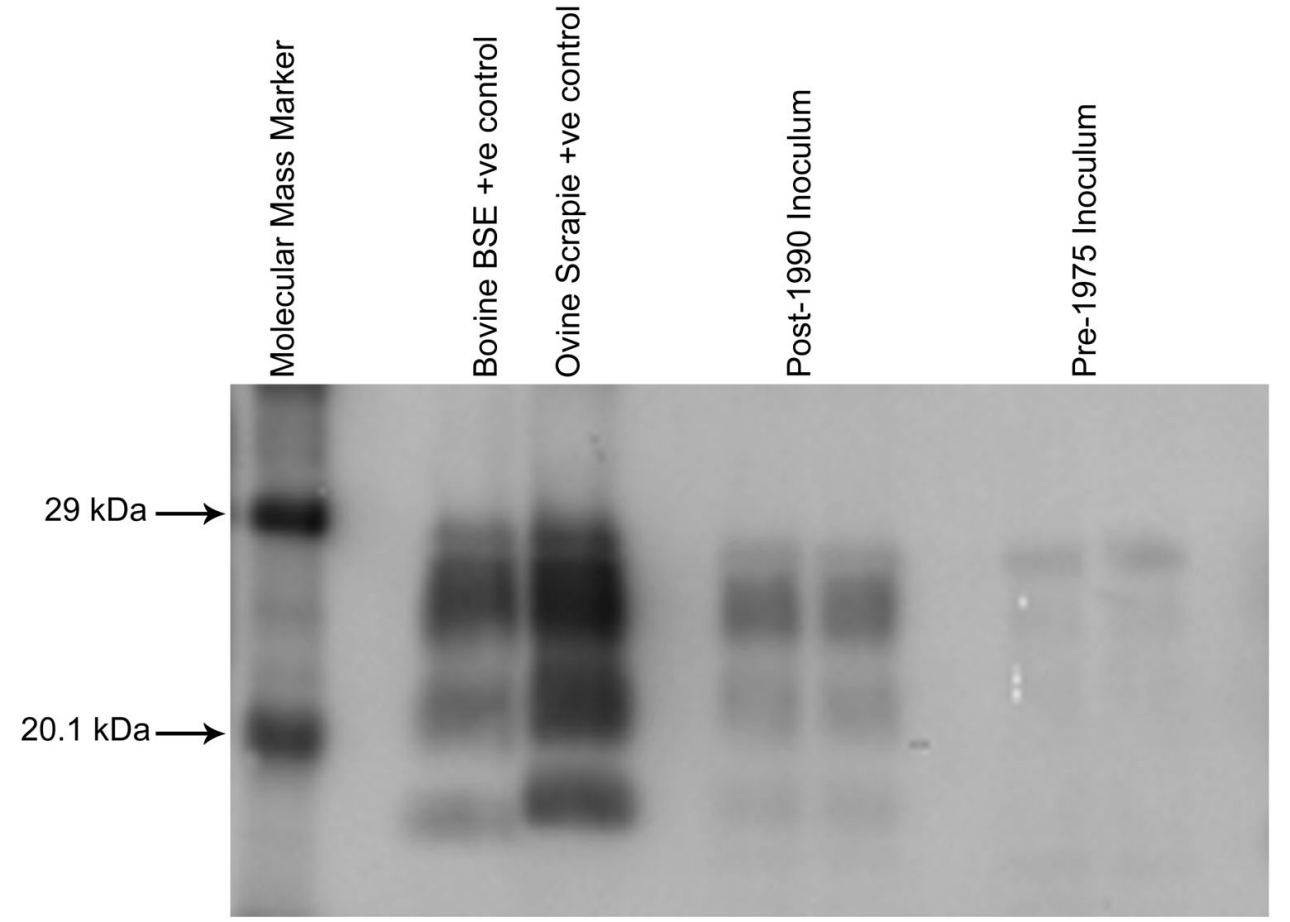
Western immunoblot with monoclonal antibody 6H4 on the original pre-1975 and post-1990 pools.

**Figure 8:**
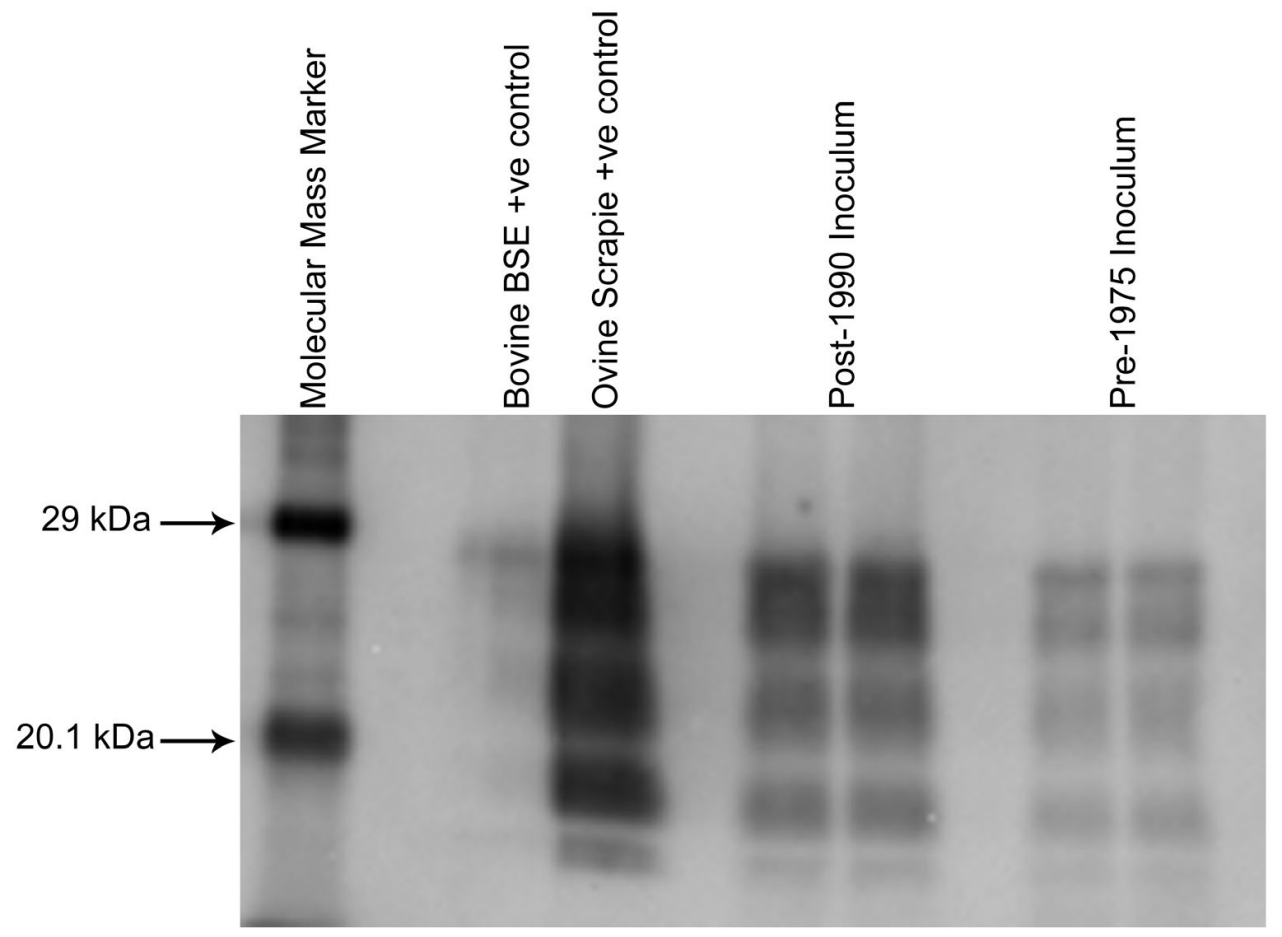
Western immunoblot with monoclonal antibody P4 on the original pre-1975 and post-1990 pools.

The WB of the original pools (pre-1975 and post-1990) gave a detectable molecular profile with mAb 6H4 only for the post-1990 pool, which was similar to the ovine scrapie control sample (Figure 7), whilst the molecular profile with mAb P4 (clear immuno-reaction) was uniform for both inocula and similar to the ovine scrapie control sample although the signal appeared stronger for the post-1990 inoculum (Figure 8).

**Figure 9:**
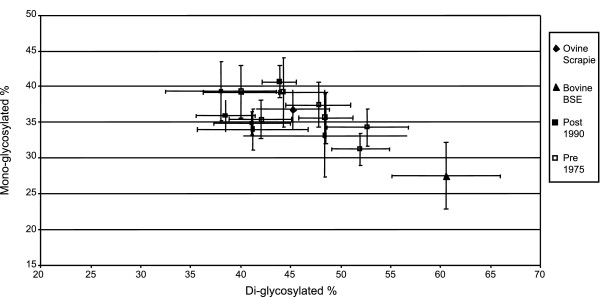
**Glycoform profiles for cattle of the pre-1975 and post-1990 group compared to scrapie and BSE**. The glycoform profiles are given as the percentage signal (mean with standard error) of the diglycosylated protein band plotted against that of the monoglycosylated band. The outlier PG152/02 of the post-1990 group is included.

**Figure 10:**
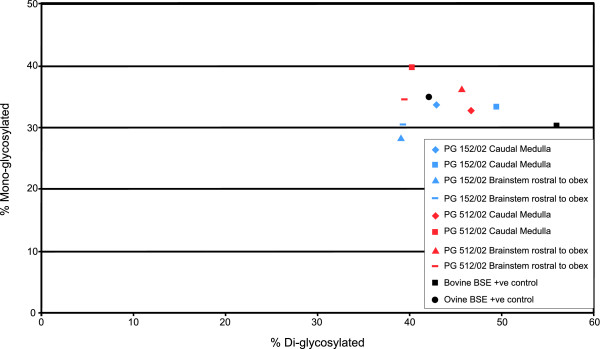
**Glycoform profiles for the two outliers of the post-1990 group compared to scrapie and BSE**. The glycoform profiles are given as the percentage signal (mean with standard error) of the diglycosylated protein band plotted against that of the monoglycosylated band. Outliers were PG152/05 and PG512/02, which gave a molecular mass profile with mAb 6H4 that was different from the other cattle in the post-1990 group.

The mean glycoform ratios obtained from individual brain samples from cattle of the pre-1975 (n = 7) and post-1990 (n = 6, included is the outlier PG152/02) group were compared to those obtained from a natural BSE case and a natural scrapie case (Figure 9). The results show that the glycoform ratio for the BSE control field sample (60.5:27.5) was separate from those obtained for the natural sheep scrapie control sample (45.2:36.8) and all other samples from the two inoculated groups (ranging between 52.7:34.3 and 38.0:39.3). The glycoform ratios for caudal medulla and brainstem tissues from the outliers of the post-1990 group (PG152/05 and PG512/05) were separate from the BSE control and were clustered close to the ratio obtained for the sheep scrapie control (Figure 10).

### Mouse bioassay

Attack rate and incubation periods in mice inoculated with the original scrapie pools and with brains from scrapie-affected cattle are given in Table 4. Transmission of the pre-1975 ovine scrapie brain inoculum into mice was successful in RIII, C57Bl and VM mice whilst the post-1990 inoculum did not transmit sufficiently into mice to determine a lesion profile. When the brains from six scrapie-affected cattle from the pre-1975 challenge group were inoculated into mice, only three transmitted sufficiently to RIII mice to determine a lesion profile. There was also insufficient transmission in C57Bl or VM mice to determine a lesion profile.

Brains from three scrapie-affected cattle from the post-1990 group that were inoculated into mice transmitted to RIII (from three steers), VM (from one steer) and C57Bl mice (from one steer).

**Figure 11:**
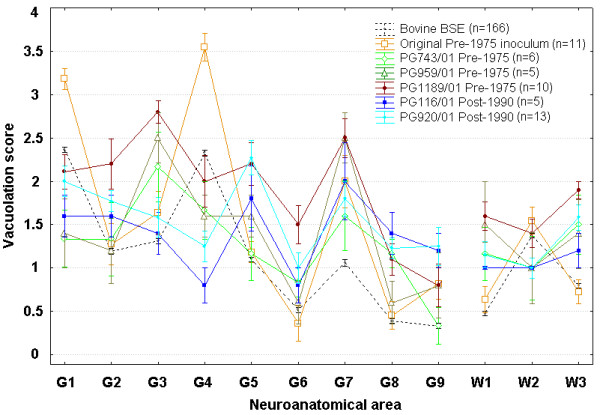
**Average lesion profiles in RIII mice for each inoculum and comparison with BSE**. G1 Dorsal medulla nuclei. G2 Cerebellar cortex. G3 Superior colliculus. G4 Hypothalamus. G5 Central thalamus. G6 Hippocampus. G7 Lateral septal nuclei. G8 Cerebral cortex (at the level of the thalamus). G9 Cerebral cortex (at the level of the septal nuclei). W1 Cerebellar white matter. W2 Tegmentum. W3 Pyramidal tract.

The lesion profiles in RIII mice and their comparison with BSE are given in Figure 11. Similarities with respect to the shape of the profiles are seen between the three cattle of the pre-1975 group (peaks at grey matter areas 3, 5 and 7), between the two cattle of the post-1990 group (peaks at grey matter areas 5 and 7) and between the original pre-1975 inoculum and the average BSE in mice (peaks at grey matter areas 1, 4 and 7), although for BSE the average lesion scores in the hypothalamus and lateral septal nuclei were considerably lower (approximately half) compared to the pre-1975 inoculum. Cluster analysis clearly separated individual cattle of the pre-1975 or post-1990 group from the original pre-1975 inoculum and natural BSE. The differences, expressed as Euclidian distances, were much smaller between the different BSE pools than between BSE and the pre-1975 inoculum (Figure 2B).

The lesion profiles of the pre-1975 inoculum and the brains of two steers of the post-1990 inoculum, which transmitted in VM mice and C57Bl mice respectively, did not resemble those of BSE with peaks of vacuolation distributed in different grey matter areas when compared to the BSE profile (data not shown).

## Discussion

Inoculation of cattle with two pools of scrapie isolates, sourced in GB before and during the BSE epidemic, resulted in transmission of two main disease phenotypes, one from each pool, as determined by neuropathological and Western immunoblotting methods as well as biological characterisation on transmission to mice. Neither of the pools transmitted a disease identical to BSE.

Previous work [11,12,15] has demonstrated that cattle are susceptible to scrapie by parenteral exposure routes and succumb to a relatively uniform neurological disease that is different to BSE in its clinical, histopathological and immunohistochemical features. These studies used scrapie isolates from the USA and it was not known if the disease was reproducible with scrapie isolates from GB.

Reported clinical signs in the recipient cattle were predominantly locomotor deficits resulting in difficulty rising and dullness or somnolence [11,12] whilst locomotor deficits in BSE are generally accompanied by over-reactivity to external stimuli [16,17]. The clinical disease produced in this study by challenging cattle with the pre-1975 inoculum resembled that described in the previous studies, but two animals challenged with the post-1990 inoculum displayed progressive nervousness with tremor and over-reactivity to external stimuli. These behavioural and sensory changes are observed in BSE cases, both in natural [16,17] and experimental [18,19] disease. As the clinical presentation between different animals can be quite variable despite similar widespread vacuolation or PrP^Sc ^accumulation in the brain, none of these neuropathological markers may be directly responsible for the observed clinical signs.

Differences between the pre-1975 and post-1990 group were also observed neuropathologically: neuronal PrP^Sc ^accumulation dominated in the pre-1975 group whilst the post-1990 group showed predominantly neuropil PrP^Sc ^accumulation, although the distribution of vacuolar pathology in the brain was similar in both groups. Only minimal vacuolar pathology was reported in two of the USA studies [11,12] despite neuronal and neuropil PrP^Sc ^accumulation [12], whereas in most clinical cases of BSE neuropil vacuolation is prominent with the greatest vacuolar densities in the medulla, midbrain and thalamus [5,6,20]. This prominence of neuropil vacuolation in the hind brain is a feature in common with the brains of cattle inoculated i.c. with the BSE agent (Figure 1), but in i.c. inoculated cattle, and indeed, laboratory rodents similarly inoculated, the fore brain areas have higher vacuolation scores compared to natural exposures (Figure 1) or peripheral routes of experimental exposure consistent with the direct route of inoculation. That this difference does not reflect divergence from the basic BSE profile is supported by the cluster analysis, which linked naturally infected BSE cases with intracerebrally BSE infected cattle. In contrast, in the scrapie-inoculated animals of both groups there was relative sparing of the BSE target areas – the nucleus of the solitary tract and the nucleus of the spinal tract of the trigeminal nerve. A comparison of this area in the two challenged groups, with cattle i.c. challenged with BSE, and a small number (n = 9) of natural cases of BSE revealed that involvement of the DNV is no greater in the scrapie-challenged animals than in BSE (M.M. Simmons, unpublished data).

Despite differences in the PrP accumulation between both scrapie-challenged cattle groups, neither of the immunolabelling patterns, or that reported in the USA study in which IHC was performed [12] resembled that of BSE [6,21,22]. The distinctive immunolabelled plaques reported in two cases of the apparently novel, naturally occurring bovine TSE in Italy [23] were not a feature of the present or previous experimental transmissions of scrapie to cattle.

WB examination of the original brain pools from scrapie-affected sheep gave a molecular profile suggestive of ovine scrapie with an obvious immuno-reaction with mAb P4. The pre-1975 inoculum was not detected with mAb 6H4. A similar finding is occasionally made in scrapie cases with pre-clinical or early clinical disease where PrP^res ^is only detected by mAb P4 (M. Chaplin, unpublished data). Both inocula comprised cerebral cortex of scrapie-affected sheep, which is less ideal for the discriminatory WB than brainstem [24]. The distribution of PrP^Sc ^or PrP^res ^within the brain of scrapie cases can vary significantly, with less PrP accumulation in the cerebral cortices [25], which has also been observed in clinical disease [26,27]. It is possible that disease-associated PrP was sparser in the cerebral cortices of sheep in the pre-1975 pool, which is supported by the weaker signal obtained with the P4 antibody compared to the post-1990 pool. Irrespective of these findings, they did not explain the different molecular profiles obtained after transmission into cattle.

The molecular profile obtained from the pre-1975 challenged cattle did show similarities to results obtained previously for experimental BSE in sheep samples, and the CH1641 sheep-passaged scrapie strain, although in both of these a weak immuno-reaction occurs with the mAb P4 [10]. Therefore, the overall molecular profile for BSE from cattle challenged with the pre-1975 scrapie pool was markedly different to that obtained for natural scrapie cases, and not identical to that obtained for experimental BSE in sheep, CH1641, or bovine BSE cases. The molecular profile for the post-1990 group was dissimilar to that obtained for cattle BSE, which gives no immuno-reaction with the mAb P4 under the test conditions. The molecular profiles obtained for the majority of the post 1990 group were more akin to those obtained for natural scrapie, but not identical, and were markedly different to those obtained for experimental BSE in sheep, CH1641, or bovine BSE cases.

The basis for discrimination by the WB test, using the two different mAbs, is the location of the N-terminal cleavage site for proteinase K digestion of PrP^res ^between BSE and scrapie. A study, using antibody blocking techniques and Pepscan analysis, suggests that the N-terminal amino acid sequence WGQGGSH remains intact only in sheep scrapie digests and is detected by the mAb P4 while it is truncated in sheep BSE and is therefore not detected by the same antibody unless it is used at high concentrations [28]. It is our experience that the P4 antibody gives only a weak immuno-reaction for PrP^res ^from experimental BSE in sheep samples and the sheep passaged scrapie strain CH1641, and no immuno-reaction with PrP^res ^from bovine BSE with this technique [10]. Interestingly, the pre-1975 group of tissues showed more similarities to BSE than the post-1990 group with regard to molecular profiles using the WB discriminatory criteria of immuno-reactivity and molecular mass. However, the molecular masses of the three PrP^res ^bands were slightly, but reproducibly, different between the pre-1975 group and the BSE control (Table 2). The values for the monoglycosylated and diglycosylated were significantly lower; therefore an exact match could not be established.

The higher molecular mass profile obtained in five of the cattle in the post-1990 group (Figure 4 and Table 2) has also been observed in brain tissue from Canada, sourced from elk and white-tailed deer affected by chronic wasting disease (CWD) and in a limited number of sheep from Canada affected by scrapie [29,30]. The molecular mass for the unglycosylated protein band obtained for the two remaining animals from the post-1990 group was lower than that obtained for the scrapie control sample, and the other five steers in the group. This is more indicative of BSE, but both animals gave marked immuno-reactions with the mAb P4, which is more indicative of scrapie, so again there was no exact match to scrapie or BSE from any of the post-1990 group.

The different molecular profile obtained on a sample of medulla rostral to the obex for PG512/02, which was the same as that of the five other steers in the post-1990 group, is of particular interest. Samples from the caudal medulla of scrapie-affected sheep in GB generally give a uniform molecular mass profile, which is independent of breed, geographic area and PrP genotype, with the WB technique used in this study [24] but it could be hypothesised that molecular mass variation in different brain regions from the same animal may be due to a particular strain or to a mixture of strains. Alternatively, differences in the degree of glycosylation of PrP^res ^found in different brain regions in mice have been attributed to different degrees with which various cell types distributed throughout the brain may glycosylate PrP^res ^[31]. This has also been hypothesised for ovine TSEs [32]. One study [29] found that the PrP^res ^glycoform pattern did not differ when six different brain regions of clinically affected deer or elk with CWD were analyzed. However, two types of PrP^res ^have been detected separately in some brain areas of cases of sporadic Creutzfeldt-Jakob disease (CJD) [33-36], which were associated with different immunohistochemical features (diffuse PrP^Sc ^deposits or plaques) that may result in different digestion properties with proteinase K [36]. This is supported by the immunohistochemical pattern in these two steers, which resembled that of the pre-1975 group in most of the brain areas examined, although a mixed pattern (resembling both the pre-1975 group and the other steers in the post-1990 group) was observed in more rostral brain sections. By contrast, variant CJD has not shown any regional variation in PrP isotype or glycosylation pattern when antibodies that detect both molecular type 1 and type 2 were used, which is suggestive of exposure to a single strain of agent [34]. Thus, the inoculation of cattle with a pool of scrapie isolates of possibly various strains may have contributed to the heterogeneity in the molecular PrP^res ^profiles observed in this study. There was no evidence that the PrP genotype of the steers was responsible for the phenotype diversity because the disease produced in the two steers (PG920/01 and PG 959/01) that differed from the other steers with respect to the octapeptide region was similar in the neuropathological and molecular characteristics to that produced in other steers in their group.

Electrophoretic molecular profiles of PrP^res ^in BSE-affected animals and humans with variant CJD have all demonstrated a lower molecular mass for the unglycosylated protein band [10,37]. Thus, in this respect, experimental BSE infections of mice [38,39] and experimental BSE in sheep [10,28,40-42], are in contrast to most other TSEs in these species. However, more recent studies in transgenic mice expressing the human prion protein have shown that the cattle BSE agent could propagate with either vCJD-like or sporadic CJD-like properties [43]. Also, it has been shown that even in vCJD and BSE two types of PrP^Sc ^can co-exist [44]. Since there has been some more direct evidence of possible different phenotypes of BSE. Two cases from Italy have been described as having novel BSE molecular and neuropathological characteristics [23]. Data from two BSE cases in young animals (23 and 21 months) in Japan have also been reported as giving different biochemical characteristics to previous cases in Japan [45]. In France, another different phenotype has also been described [46]. In this last study a new phenotype was described, in three samples out of 58 French BSE cases tested, which exhibited a higher molecular mass of unglycosylated PrP^res ^and were clearly reactive with mAb P4. As this was the predominant molecular profile found for the post-1990 group in this present study it could indicate that the scrapie pool used for the inoculation of this group may have contained a different strain, agent or particular cellularly located PrP^Sc ^that was different to that in the pool used for the pre-1975 inoculations, and which manifested as a different phenotype in cattle, even though the original post-1990 pool gave a uniform scrapie-like profile. The finding of significantly different PrP^res ^profiles for sporadic CJD and variant CJD has led to speculation of an analogous spontaneous rare sporadic form of BSE in cattle that might even have been the origin of the BSE epidemic [37,41,47].

Previously, glycoform ratio analysis has been used to aid in discrimination between experimental cases of BSE in sheep and scrapie in sheep [8]. However, there is considerable variation in signal when individual samples are repeatedly measured, which leads to large standard error measurements in the glycoform ratio analyses. In this previous research the glycoform ratios obtained for natural scrapie cases and bovine BSE cases could not be distinguished whereas experimental BSE in sheep ratios were distinct from natural sheep scrapie. In follow-up studies on the reproducibility of the VLA hybrid test with regard to molecular masses and glycoform ratio, the precision of the technique was satisfactory for the molecular masses, with coefficients of variation of less than 3%. This was not the case for the glycoform measurements. When the signal density for each single band was analysed repeatedly from the same sample the coefficients of variation exceeded 10% and the variation for the glycoform ratio of the diglycosylated band: monoglycosylated band was 26% (M. Stack, unpublished data). Therefore, unless the differences are large it is clear that glycoform ratio analysis is a less useful tool for discriminating between bovine BSE and scrapie. However, in this study the glycoform ratios of all samples, regardless of the inoculum or molecular weight profile, were clustered around the ratio obtained for the natural sheep scrapie control and separate from the ratio obtained for the BSE field case (Figures 9 and 10).

Our results suggest that different forms of scrapie can affect cattle and that resultant prion diseases in cattle may have different manifestations, which might not be recognised as readily as typical BSE when transmitted to other species such as sheep. These possibilities increase the importance of further investigation into pathological and biochemical profiling for TSEs in ruminant species and in fact a study is now underway in the UK which will examine tissue from retrospective BSE cases for possible signs of phenotype variation not detected by earlier diagnostic methods.

Conventional strain typing assays confirmed the transmission of a prion disease in recipient cattle. The mouse lesion profiles obtained from the original inocula and after passage through cattle were different for both groups, which is not unexpected since there was most likely a mixture of different scrapie strains in the pools. Nonetheless, the profiles were unlike those obtained with BSE. The original pre-1975 inoculum gave a lesion profile and incubation period range in RIII mice with some similarities to BSE although the lesion profile peaks were greater than those for BSE (Figure 11). In addition, cluster analysis revealed that different BSE pools shared more similarities (measured as Euclidian distance) with each other than with the original pre-1975 inoculum (Figure 2B). Similar profiles in RIII mice have been obtained from individual scrapie isolates sourced from the UK although they subsequently differed from BSE on second passage in mice (R. Green, unpublished data). In addition, the generally poor attack rate in RIII mice after inoculation of brains of scrapie-affected cattle is unlike BSE. This is in agreement with the US study where the brain from a clinically affected cow infected with scrapie failed to transmit to two mouse strains, NIH Swiss-Webster and NZW/LacJ [48]. Besides, the lesion profiles in the other two mouse strains (VM and C57Bl) obtained after inoculation with the original pre-1975 inoculum did not resemble BSE.

Parenteral challenges of cattle with BSE affected brain homogenate have, in general, produced a higher attack rate (100%) and shorter incubation periods [49] when compared to the cattle challenges with scrapie material in this study. Clearly, this is, in part, related to dose, both with respect to amounts and titer of inocula as shown by decreasing attack rate and decreasing incubation period when cattle are i.c. inoculated with BSE brainstem using a 10-fold dilution range from 10^-3 ^to 10^-8 ^(Hawkins SAC, Wells G, Austin A, Ryder S, Dawson M, Blamire I, Simmons M: **Comparative efficiencies of the bioassay of BSE infectivity in cattle and mice**. In Proceedings of the Cambridge Healthtech Institute's 2^nd ^International Transmissible Spongiform Encephalopathies Conference: 2–3 October 2000; Alexandra, Virginia, USA). Nevertheless, the inocula were prepared from cerebral cortices of clinically affected scrapie cases and would be expected to contain a high concentration of infectious agent, albeit slightly lower than in the brainstem [50]. This relatively low transmissibility may suggest that scrapie strains have to undergo some adaptation process, perhaps requiring serial passage, to survive as pathogens in the cattle population. The historical use of meat and bone meal would have provided this large potential for passage through cattle.

In addition, little is understood regarding host responses to infection with a mixture of TSE strains of different pathogenicity. Molecular analysis has demonstrated that co-infection of mice with a mouse-adapted scrapie and BSE strain results in a scrapie-like molecular profile [38]. A mixture of ovine brains affected by scrapie and BSE inoculated into RIII, C57Bl or VM mice resulted in a mouse lesion profile unlike BSE on first passage [51] although the BSE strain usually produces a shorter incubation period and a higher attack rate in RIII mice than scrapie isolates [52]. Thus, the natural infection of cattle with various scrapie strains, some of which might be more pathogenic to cattle, may not produce a BSE-like disease on first passage. The use of transgenic mouse rodent models may further aid in the interpretation of strain characteristics of isolates from this study.

This study does not support the hypothesis that BSE was caused by the replication of a scrapie strain in cattle, but it was based on the two scrapie pools, the individual sheep agent strain content of which was not characterised (due to insufficient material) and which may not have been representative of the spectrum of scrapie strains that may have existed in the GB sheep population. Also, the presence in the pools of a scrapie strain that may produce a BSE-like disease when transmitted to cattle cannot be excluded since strain characteristics may also change after transmission to other species. Thus, further sub-passage of brains from scrapie-affected cattle into cattle is desirable to investigate the effects of passage on the stability of the phenotypes defined on primary transmission. Second passages of the scrapie agent in cattle in studies conducted in the USA have demonstrated that the appearance and topographical distribution of neurohistological changes and/or the distribution of PrP^Sc ^was almost identical to that produced by primary inoculation [13,14]. Interestingly, the description of clinical signs resembled BSE in one passage study [14]. The studies in the USA have produced a relatively uniform neurological disease in cattle after inoculation with several different sources of pooled scrapie-infected tissue. This differed from BSE in cattle with respect to both clinical and histopathological characterisation. The findings in this study do not suggest the same degree of uniformity in the disease presentation produced by inoculation of two different pooled inocula. Neither of these presentations are identical to BSE but share some aspects of characterisation in common. While the characterisation of the disease forms in the cattle did not reflect an entirely uniform phenotype after inoculation with the post-1990 pooled scrapie source, it is of interest in relation to the phenomenon of selection of agent strains from a mixture on transmission across a species barrier [53], that the phenotype within the pre-1975 pooled scrapie recipients was uniform and that this phenotype also occurred within recipients of the post-1990 pooled scrapie source. Whether differences between the phenotypes detected in the temporally separated scrapie source pools is simply related to TSE agent strain diversity within the sheep population, or has some epidemiological significance in terms of changes in extant agent strains over time, cannot be determined from the present study.

## Conclusion

Cattle infected with sheep scrapie agents may develop a disease that is different from BSE in its clinical, pathological and biochemical features and variable disease presentations may exist. The process by which scrapie strains might produce a BSE-like disease in cattle was not evident from this primary transmission experiment. However, it is diagnostically significant that despite differences in the biochemical and immunohistochemical characteristics within both scrapie-infected groups in this study, and between the 'bovine scrapie' phenotypes and BSE, a prion disease was nevertheless diagnosed by IHC and WB examination of brainstem samples, consistent with internationally approved prion disease diagnostic methods in ruminant species.

## Methods

All procedures involving animals were approved by the Home Office under the Animals (Scientific Procedures) Act 1986.

### Inocula

Two pools of homogenised brain were prepared as described previously [54] from frozen archived cerebral cortex of sheep of several different breeds affected with natural scrapie, which was confirmed by histopathological examination of the brain. The first comprised material from eleven sheep killed prior to 1975 (five Cheviot, four Suffolk and two Shetland) and the second, material from ten sheep killed after 1990 (six Cheviot, two Suffolk, one Halfbred and one Herdwick). Each scrapie case contributed approximately 1 g of brain material to the pools. The sourcing periods were chosen to reflect the situation in GB, where the occurrence of BSE had been unlikely prior to 1975 whilst the epidemic was well established by 1990 and still increasing over the next two years. Brain material from ten sheep (five Cheviot, one Dorset, four Suffolk) imported from New Zealand, which is free from scrapie, was prepared similarly as a TSE free control inoculum. Histopathological and immunohistochemical examinations of the donor brains were negative for scrapie. A DNA sequencing analysis using a cleaved amplified polymorphic sequences method [55] as well as species-specific real time polymerase chain reaction analysis [56] confirmed the sole presence of ovine DNA in the brain pools.

### Inoculation of cattle

A total of thirty castrate male Friesian/Holstein calves were acquired from farms in GB with no history of BSE, no evidence of exposure to meat and bone meal and no known cases of BSE when admitted to the study (April 1999 – March 2000). Prior to inoculation all calves received a neurological examination and were considered free from any neurological disease. Two groups of ten calves each received one of the pooled sheep scrapie inocula. At 4–6 months of age each calf was inoculated i.c. with 1.0 ml of a 10 per cent homogenate in saline of one of the scrapie brain pools. Two control groups of five age-matched castrate male cattle were similarly inoculated, one with brain from sheep free from TSE and one with sterile physiological saline solution. The i.c. inoculation method used a semi-stereotaxic technique, which ensured anatomically reproducible deposition of inoculum [54]. To prevent possible cross-contamination of animal groups, staff were required to wear protective clothing, with one set dedicated per pen, and all facilities used to examine the animals (corridor, crush) were decontaminated by exposure to 20% sodium hypochlorite solution for at least one hour between use for different groups.

PrP genotype of the calves, with respect to the octapeptide repeats, was determined as described previously [19]. Two animals (PG920/01 in the post-1990 group and PG 959/01 in the pre-1975 group) were heterozygote for six copies of the octapeptide (6:5), the remaining animals were homozygote (6:6).

### Clinical assessments

Clinical assessments comprised weekly observations for 15 minutes per group of five cattle to detect BSE-like signs or any other neurological abnormality and monthly to quarterly neurological examinations depending on clinical progression from 12 months after inoculation (for detailed description of assessment methods refer to [54]). In addition, cattle were observed on two occasions for 12 and 24 hours, respectively, to record signs of disease that may not have been evident at weekly observations. This was later replaced by Closed Circuit Television monitoring. Observations made in daily diaries by animal husbandry staff were also included to determine the clinical status.

### Postmortem investigations

Cattle that developed a progressive neurological disease were euthanased with pentobarbital administered intravenously. The brain was removed aseptically, an intact transverse section of the medulla oblongata at the level of the obex sampled into 10% formal saline and the remaining brain tissue bisected sagitally. The larger part containing the midline structures was also fixed in formal saline for neuropathological examination and the contra-lateral part frozen at -80°C. Fixed tissues were prepared for routine histopathological examination in haematoxylin and eosin (HE) stained sections and for immunohistochemical examination labelled for PrP^Sc^. Whilst the first three cases were labelled with rabbit polyclonal antibody RB486 [57], further cases were labelled with rat monoclonal antibody R145 [58]. Both antibodies are C-terminal specific, raised against the same bovine peptide sequence and are comparable qualitatively for the immunohistochemical detection of PrP^Sc ^in scrapie and BSE (M.M. Simmons, unpublished data). CNS regions examined comprised the medulla oblongata at the level of the obex, the rostral medulla at the level of the cerebellar peduncles, caudal and rostral midbrain, cerebellum, thalamus, occipital, parietal and frontal cortices as well as spinal cord segments C5, T1, T6, T12 and L5–6. As part of the histopathological examination, seventeen neuroanatomical sites of the brain were scored, on a 0–4 scale, for the severity of vacuolation in HE stained sections to determine a lesion profile [5]. Scoring was conducted without knowledge of the inoculation status of each animal and by the previously described method [5]. To maintain uniformity of observations with those of previous studies, only operators trained by M.M. Simmons carried out the scoring. The lesion profiles obtained were compared to those of naturally infected BSE cases diagnosed between 1987 and 1989 [5] and to those of ten cattle i.c. infected with BSE, which were derived from a separate study conducted at VLA Weybridge (Hawkins SAC, Wells G, Austin A, Ryder S, Dawson M, Blamire I, Simmons M: **Comparative efficiencies of the bioassay of BSE infectivity in cattle and mice**. In Proceedings of the Cambridge Healthtech Institute's 2^nd ^International Transmissible Spongiform Encephalopathies Conference: 2–3 October 2000; Alexandra, Virginia, USA). Briefly, groups of male castrate calves were each inoculated i.c. at approximately 4 months of age with a single dilution of inoculum prepared from pooled brainstems from BSE affected cattle using a ten fold dilution range of 10^-3 ^to 10^-8^. Cattle were monitored clinically until they developed neurological signs consistent with BSE when they were killed and the brain examined to confirm the morphological phenotype of BSE and the presence of disease specific PrP by IHC. The lesion profiles for comparison with scrapie in cattle were obtained from brains of ten BSE-affected cattle selected from the various inoculation groups (10^-3 ^– three animals, 10^-4 ^– two animals, 10^-5 ^– one animal, 10^-6 ^– two animals and 10^-7 ^– two animals). The average lesion profiles in cattle were compared by cluster analysis of the average lesion score in each neuroanatomical area and the results displayed as vertical hierarchical tree plots as described for strain characterisation of ovine scrapie [52]. Similarities of the profiles were measured as Euclidean distance (Statistica 7.0 Statistical software, StatSoft Inc., USA).

For the immunohistochemical examination, the CNS regions were assessed for the localisation (neuropil or neuronal) and type (particulate, linear, stellate) of PrP^Sc ^immunolabelling [6].

Frozen tissues from the caudal medulla were examined by a WB technique developed at the Veterinary Laboratories Agency (VLA) to allow the comparison of molecular profiles ('VLA hybrid technique', [8,10]). PrP^res ^is detected by WB techniques as three protein bands which relate to diglycosylated, monoglycosylated and unglycosylated forms of the abnormal protein [59]. Differences between the molecular masses of the unglycosylated protein band of PrP^res ^in natural scrapie, cattle BSE, and experimental BSE in sheep cases has been previously published for this technique [10]. Discrimination was also possible by parallel testing using two specific mAbs. The first, mAb 6H4 (Prionics AG, Schlieren, Switzerland) is a mouse IgG1 antibody raised to human PrP, and was shown to have affinity to the linear epitope amino acid sequence 144–152 [60], and this sequence is equivalent to the 155–163 amino acid sequence of bovine PrP and the sheep PrP sequence 147–155 [61], and therefore detects PrP^res ^in both cattle and sheep [30]. The second, mAb P4 (Biopharm, Darmstadt, Germany), is raised to a peptide with amino acid sequence of ovine PrP 89–104 and is more selective for scrapie PrP^res ^under our test conditions due to its affinity to ovine epitope amino acid sequence 93WGQGGSH99 [28,62] which differs from the bovine sequence at one position 101WGQGGTH107.

As there were cases with an immunoblotting profile that differed from that of other cases in the same inoculation group, a second sample of the brainstem taken slightly rostral to the obex was also examined, but for the purposes of this report these examinations were limited to five cases of the pre-1975 group and three cases of the post-1990 group to ensure that enough material was available from other animals for future use.

Positive control tissue for the WB was obtained through normal routine submissions of ovine and bovine brain to the VLA. The natural scrapie control sample was from a Shetland/Cheviot cross bred sheep with a PrP^ARQ/ARQ ^genotype. The bovine BSE sample was from a clinical suspect case that was positive by statutory diagnostic methods (histopathological examination of medulla (obex) with a typical vacuolar distribution pattern and WB for PrP^res ^using the Prionics^®^-Check WESTERN). All samples except the bovine BSE and ovine scrapie positive controls were run in duplicate in adjacent lanes of the gels and the mean values for the unglycosylated, monoglycosylated and diglycosylated molecular masses were used in the statistical analyses of the variance, followed by tests of pre-specified contrasts among the group means.

The immunoblots were visualised by means of enhanced chemiluminescence (CPD-Star, Tropix). The blots were quantified (molecular mass measurements and relative quantity of the signal for glycoform analysis) using Fluor S Multimager computer analysis (Quantity One, software Biorad UK Ltd). For these analyses the centre position of each protein band is used as the reading point. For glycoform analysis the combined signals from the blots treated with mAb 6H4 are defined as 100% and the contribution of each band calculated as a percentage of the whole. The ratio of the mean values of the high molecular mass glycoform (diglycosylated band) and the low molecular mass glycoform (monoglycosylated band) are plotted as a scattergraph. The percentage signal obtained from the unglycosylated band (the lowest) is not used. The mean value and standard errors were obtained from three duplicate gels for samples from the inoculated groups, pre-1975 and post-1990, and for the BSE and scrapie controls.

The discriminatory WB (VLA Hybrid technique) was applied retrospectively to the original scrapie pools (brain homogenate as 10^-1 ^dilution) to allow comparison with the blots obtained from scrapie-affected cattle.

### Mouse bioassay

The original sheep scrapie brain pools and brainstems from cattle that developed a prion disease (six from the pre-1975 group, three from the post-1990 group) were each inoculated into a standard panel of three isogenic mouse strains (RIII, C57Bl/6J, VM) for biological strain typing. Mouse groups consisted of twenty mice. Both mouse strains RIII and C57Bl carry the 'a' allele of the PrP gene, which results in a shorter incubation period, whilst the VM strain carries the 'b' allele of the gene; the alleles encode proteins that differ by two amino acids at codons 108 and 189 [63]. These mouse strains had been used before at VLA to characterise BSE and scrapie isolates. The methods of inoculation and lesion profiling have been described previously [4]. Transmission was sufficient to determine a lesion profile if at least five mouse brains were affected by spongiform changes [64]. The average lesion profiles in RIII mice for each inoculum were compared by cluster analysis as described above. Ten different brain pools (natural BSE pools 1901) from a total of 197 naturally infected BSE cases, which had been prepared for a BSE pathogenesis study [19] and inoculated into RIII mice (published previously [4]), were used for comparison of the scrapie inocula with BSE.

## Authors' contributions

TK carried out the clinical assessments and managed the study. YHL, MMS and JS carried out the histopathological and immunohistochemical examination of the bovine brains, whilst CH and RBG carried out the histopathological examination of the murine brains. MJS and MC were responsible for the Western immunoblot examination. SACH, JWW and GAHW participated in the design, initiation and coordination of the study. RL prepared the inocula and provided the mouse attack rate data. All authors contributed to the final manuscript.

## Figures and Tables

**Table 1:** Immunolabelling pattern by brain area

**Case No**	**Group**	**Clinical** **presentation**	**Obex**	**Rostral** **medulla/** **Cerebellum**	**Rostral** **midbrain**	**Rostral** **thalamus**	**Basal** **ganglia/** **frontal** **cortex**	**Antibody**
119/01	Pre 1975	Dull form	1	1 (Cerebellum negative)	1	1	1	486
609/01	Pre 1975	Dull form	1	1	1	1	1	486
743/01	Pre 1975	Dull form	1	1	1	1	1	145
749/01	Pre 1975	Dull form	1	Negative	1	1	1	145
959/01	Pre 1975	Dull form	1	1	1	1	1	145
1189/01	Pre 1975	Dull form	1	1	1	1	1	145
045/02	Pre 1975	Dull form	1	1	1	1	1	145
754/02	Pre 1975	Dull form	1	1	1	1	1	145
166/04	Pre 1975	Dull form	1	1	1	1	1	145
116/01	Post 1990	Dull form	2	2	3	3	3	486
920/01	Post 1990	Nervous form	2	2	3	3	3	145
1063/01	Post 1990	Dull form	2	2	3	3	3	145
152/02	Post 1990	Dull form	1	1	1	1+3	1+3	145
512/02	Post 1990	Nervous form	1	1	1	1	1+3 (Cortex negative)	145
1215/02	Post 1990	Dull form	2	2	3	3	3	145
96/04	Post 1990	Dull form	2	2	3	3	3	145

Group 1 – Predominantly neuronal with less neuropil labelling

Group 2 – Predominantly neuropil with less neuronal labelling (in both groups the overall distribution of IHC types is similar)

Group 3 – Like group 2 but with prominent aggregated/stellate forms

**Table 2:** Comparison of the mean molecular masses (kDa) between the control samples and the two scrapie-inoculated groups

**Group**	**No. of samples (of n animals)**	**Unglycosylated Mean (SEM)**	**Monoglycosylated Mean (SEM)**	**Diglycosylated Mean (SEM)**
BSE control	4 (n = 2)	18.6 (.12)	22.1 (.14)	26.3 (.16)
Scrapie control	4 (n = 2)	18.8 (.12)	22.2 (.14)	26.4 (.16)
Pre-1975 ^1^	14 (n = 7)	18.4 (.05)	21.8 (.05)	25.8 (.06)
Post-1990 ^2^	10 (n = 5)	20.3 (.06)	23.7 (.06)	27.1 (.08)

^1 ^Not included are two animals with insufficient brain material for a comparative analysis.

^2 ^Not included are the two animals that had a profile similar to the pre-1975 group.

**Table 3:** Mean differences (kDa) and the 95% confidence intervals between the pre-1975 group (n = 7) and the post-1990 group (n = 5)

**Protein Band**	**Mean difference**	**95% confidence interval**
Unglycosylated	1.83	1.68 – 1.98
Monoglycosylated	1.91	1.74 – 2.08
Diglycosylated	1.31	1.11 – 1.52

**Table 4:** Attack rate and mean incubation periods in mouse strains after transmission of ovine scrapie and bovine scrapie

	**Mouse strains**	**RIII**		**C57Bl**		**VM**	
		**AR**	**IP, SEM**	**AR**	**IP, SEM**	**AR**	**IP, SEM**

**Pre-1975 group**	**Pre-1975 pool**	11/11	477, 9	17/17	562, 13	13/16	698, 17
	**PG119/01**	3/4	898, 56	1/8	726	1/6	741
	**PG609/01**	4/6	904, 21	0/20		0/20	
	**PG743/01**	7/14	866, 45	1/8	785	2/9	754, 90
	**PG959/01**	5/13	801, 53	0/20		0/20	
	**PG749/01**	8/13	937, 36	0/20		1/15	585
	**PG1189/01**	10/11	918, 14	6/19	769, 58	3/4	839, 8

**Post-1990 group**	**Post-1990 pool**	3/12	614, 20	4/18	603, 48	1/2	797
	**PG116/01**	7/16	587, 29	0/20		0/20	
	**PG920/01**	13/16	576, 27	7/18	643, 46	0/20	
	**PG1063/01**	0/20		1/14	728	5/8	776, 20

AR Attack rate (calculated as the proportion of mice that developed disease out of the total number of mice still alive at the first positive diagnosis)

IP	Mean incubation period [days]

SEM Standard error of mean [days]
